# Effect of Larval Nutrition on Maternal mRNA Contribution to the *Drosophila* Egg

**DOI:** 10.1534/g3.118.200283

**Published:** 2018-04-17

**Authors:** Amanda E. Crofton, Emily L. Cartwright, Anna A. Feitzinger, Susan E. Lott

**Affiliations:** Department of Evolution and Ecology, University of California, Davis CA 95616

**Keywords:** maternal mRNA deposition, effects of nutrition, gene expression, life history, RNA-Seq

## Abstract

Embryonic development begins under the control of maternal gene products, mRNAs and proteins that the mother deposits into the egg; the zygotic genome is activated some time later. Maternal control of early development is conserved across metazoans. Gene products contributed by mothers are critical to many early developmental processes, and set up trajectories for the rest of development. Maternal deposition of these factors is an often-overlooked aspect of parental investment. If the mother experiences challenging environmental conditions, such as poor nutrition, previous studies in *Drosophila melanogaster* have demonstrated a plastic response wherein these mothers may produce larger eggs to buffer the offspring against the same difficult environment. This additional investment can produce offspring that are more fit in the challenging environment. With this study, we ask whether *D. melanogaster* mothers who experience poor nutrition during their own development change their gene product contribution to the egg. We perform mRNA-Seq on eggs at a stage where all mRNAs are maternally derived, from mothers with different degrees of nutritional limitation. We find that nutritional limitation produces similar transcript changes at all degrees of limitation tested. Genes that have lower transcript abundance in nutritionally limited mothers are those involved in translation, which is likely one of the most energetically costly processes occurring in the early embryo. We find an increase in transcripts for transport and localization of macromolecules, and for the electron transport chain. The eggs produced by nutrition-limited mothers show a plastic response in mRNA deposition, which may better prepare the future embryo for development in a nutrition-limited environment.

The earliest stages of embryonic development are entirely dependent on maternally deposited RNAs and proteins, until the zygotic genome is activated later in development (Tadros and Lipshitz 2009; Schier 2007; Langley *et al.* 2014). While the length of the maternally driven portion of early embryogenesis varies among species, both in absolute and relative time of development, the maternal genetic control of early development that sets up trajectories for the rest of development is a conserved feature across animals and some plants (Tadros and Lipshitz 2009; Yartseva and Giraldez 2015; Robertson and Lin 2015; Li *et al.* 2013; Baroux and Grossniklaus 2015). Many fundamental developmental processes are initiated by maternal factors, making the maternal contribution to early development highly critical, and therefore they have been the subject of considerable study. The composition of gene products that mothers contribute to eggs has been investigated in a number of model systems at a genomic scale (Li *et al.* 2010; Laver *et al.* 2015; Harvey *et al.* 2013).

The contribution of mRNAs and proteins to the egg by the mother is an often-overlooked aspect of parental investment in offspring fitness. The maternal provisioning of nutrients to offspring and subsequent effect on offspring life history and fitness has been examined in a number of systems (Mousseau and Fox 1998). Mothers that experience poor nutrition will have more limited resources to devote to provisioning of offspring. Thus effect of limited parental nutrition may be detrimental to the fitness of the offspring. However, mothers may be able to alter provisioning when they have experienced unfavorable environmental conditions to enhance offspring performance under the same unfavorable environmental conditions. Life history theory predicts that under stressful conditions, mothers may be expected to shift toward fewer offspring with better provisioning (Roff 1992). Provisioning is largely viewed through the lens of providing nutrition to the offspring, with egg size or offspring size used as an easily measured proxy.

Drosophila is a well-studied model system for maternal investment and life history (Lack *et al.* 2016; Porcelli *et al.* 2017; Prasad and Joshi 2003; Yadav and Sharma 2014), as well as for metabolic studies relevant to human disease (Alfa and Kim 2016; Padmanabha and Baker 2014). Poor parental nutrition in Drosophila has been demonstrated to result in a mix of potentially maladaptive and adaptive phenotypes in offspring. Flies with poor nutrition at the larval stage become smaller flies, yet lay heavier eggs (Prasad *et al.* 2003; Vijendravarma *et al.* 2010). This contrasts with the positive correlation of egg size and body size observed within species (Azevedo *et al.* 1997), which would predict that the eggs laid by smaller mothers would be smaller. As egg size is an approximation of maternal investment in offspring in species lacking parental care, this can be viewed as an increase in maternal investment per offspring in these nutritionally deprived mothers. And while all studies reported larger eggs from nutritionally limited mothers, the flies that developed from those eggs were reported to be smaller (Vijendravarma *et al.* 2010), larger (Valtonen *et al.* 2012), or the same size (Prasad *et al.* 2003) as those produced by mothers under standard nutritional conditions. Note that while these studies all limited nutrition at the larval stage for mothers, they limited nutrition in different ways, and used strains with different genetic backgrounds. The offspring of nutritionally deprived mothers also have higher viability to adulthood than mothers raised on standard food, when the offspring are raised on both standard and poor food in one study (Valtonen *et al.* 2012) and only when raised on standard food in other studies (Prasad *et al.* 2003; Vijendravarma *et al.* 2010). These results imply that the additional investment of the mother experiencing poor nutrition is beneficial to the offspring under some conditions.

In this study, we ask if and how maternal provisioning of gene products (specifically mRNAs) provided to the egg changes with poor maternal nutrition at the larval stage. We limit nutrition only at the larval stage in the parents, thus any observed differences are due only to the parental diet during development. We collected eggs from a developmental stage where all mRNAs present are maternally derived (Bownes’ stage 2), extracted RNAs, and performed mRNA-Seq. Analysis of the RNA-Seq data shows striking patterns of differential transcript deposition into eggs by nutritionally limited mothers. These mothers deposit fewer transcripts for cytoplasmic ribosomes and translation, and more transcripts for macromolecule transport and localization and for the electron transport chain. These coordinated changes in gene expression across nutrition-limited mothers do not correspond with genes previously identified as responding to nutrition, which is consistent with these individuals not being nutrition-limited at the time of egg production. Instead, we interpret these differences in expression relative to known expression patterns of these genes in oogenesis, to understand the potential benefit or detriment to the eggs, as development progresses.

## Materials and Methods

### Larval diet

To raise larvae on food with varying levels of nutritional restriction, we prepared dilutions (Vijendravarma *et al.* 2010) of a standard cornmeal food recipe. The standard food was melted and diluted with an autoclaved water-agar mixture, to contain 100% (not diluted), 25%, and 5% of the original cornmeal food. This was then portioned into bottles, with at least 2 replicate bottles per food treatment, where it solidified. Then, 50 eggs were added to each bottle, from 4-10 day old, population controlled, Oregon-R females, reared at 25 degrees C. Multiple rounds of bottles were set up, and effects on development time and fecundity were observed. The bottles used to collect eggs from were typical for these parameters. The larvae developed in the 100%, 25% and 5% bottles at different rates, with nutritionally limited flies beginning to eclose in the 25% and 5% bottles days later (2 days later for the 25%, 5 days later for the 5%).

### Sample acquisition

Female and male flies (10 each, newly eclosed) reared on 100%, 25%, and 5% food were collected and placed in an egg collection bottle, and supplied with a standard glucose-based egg-laying plate. Females were 2-14 days old at the time of egg collection. Eggs were collected from egg-laying plates (for each collection, a new plate was harvested after ∼30 min), dechorionated using 50% bleach, and embryos were moved to a microscope slide with halocarbon oil for visualization. Embryos were observed, imaged (Zeiss AxioImager M2), and harvested as they reached stage 2 (Bownes’ stages) of development (Bownes 1975; Campos-Ortega and Hartenstein 1985). At stage 2, all mRNAs present are maternally derived (Ali-Murthy *et al.* 2013). This stage is also easy to identify based on morphology, as the vitelline membrane recedes from both the anterior and posterior of the embryo, but the pole cells are not yet visible at the posterior. This allows the collection of the same morphological stage, despite any possible differences in development time between treatments.

Once imaged, total RNAs were extracted from embryos as in our previous studies (Paris *et al.* 2015; Lott *et al.* 2014; Lott *et al.* 2011). Briefly, embryos were removed from oil to Parafilm (Bemis), where the oil was removed, and the embryo was rolled into a drop of TRIzol reagent (Ambion), where the embryo was ruptured with a needle, and left to dissolve. Once dissolved, the drop of TRIzol was moved to a tube with more TRIzol, and extracted with a glycogen carrier according to manufacturer’s instructions, with the exception of using 1mL of TRIzol per embryo (which is an excess compared to the expected amount of total RNA). Protocol available upon request.

On average ∼100ng total RNA was extracted from an individual embryo. RNA quantity was measured using a Qubit 2.0 fluorometer (Invitrogen). RNA quality was assessed using an Agilent Bioanalyzer. Total RNA from three individual embryos (three replicates) for each food treatment were chosen, samples were sent to the DNA Technologies Core at the UC Davis Genome Center for mRNA-Seq library construction and sequencing. Sequencing libraries were constructed using oligo (dT) to enrich for polyadenylated transcripts. Libraries were sequenced (150bp, paired-end) pooled in a single lane on an Illumina HS 4000 sequencer.

### Data processing and differential gene expression analysis

Reads were trimmed and adapters removed using Cutadapt (Martin 2011), and gently (PHRED Q < 5) trimmed for quality (Macmanes 2014). Mapping was done with the *D. melanogaster* Ensembl genome assembly BDGP6 and associated annotation file. Reads were aligned and transcript abundances quantified (in TPM, Table S1) using Kallisto (Bray *et al.* 2016). Differential expression analysis at the transcript and gene level was conducted using Sleuth (Pimentel *et al.* 2017), and gene level abundance counts (in scaled reads per base) quantified (Table S2). Using Sleuth, we construct two models, one where transcripts/genes are at the same abundance in all samples, and the other where transcripts/gene abundances differ by food percentage, and identify transcripts/genes that have a significantly better fit the latter model using a likelihood ratio test. Using a FDR adjusted p-value (Benjamini-Hochberg) of 0.05, there were 119 significantly differentially expressed transcripts (Table S3), and 357 differentially expressed genes (Table S4) between treatments in our dataset. Kallisto allows multiple mapping, so examining the 357 genes, we find 314 genes with unique mapping counts, as the same reads map the same number of times to genes with high degrees of similarity (histone genes, snRNA:U1 genes). For all analyses here, we group all of these multiple mapping genes together (*i.e.*, all His4 genes with the same number of counts mapping are treated as a single “gene”). Most differentially expressed transcripts are represented in the differentially expressed gene list (86%, or all but 16). Transcripts that are significant at the transcript level but not the gene level are one of a number of transcripts for a given gene, and often have lower abundance than other transcripts for that gene. In contrast, the larger number of significantly differentially expressed genes than transcripts represent multi-isoform genes where the individual isoforms fail to meet statistical significance, but summing expression over isoforms at the gene level does show statistical significance.

During oogenesis and early embryogenesis, translation is regulated through a number of mechanisms, including those that act to increase (Benoit *et al.* 2008; Cui *et al.* 2008) or decrease (Barckmann and Simonelig 2013; Temme *et al.* 2014; Vardy and Orr-Weaver 2007) poly(A)-tail length of transcripts. In this stage of development, poly(A) tail length is correlated with translation rate, with increased translation of transcripts with lengthened poly(A) tails (Salles *et al.* 1994). This poses a complication for the interpretation of our data: as our sequencing libraries were constructed using oligo-(dT) enrichment, are we recovering a biased subset of the mRNAs present in the embryo? Two recent studies provide estimates of poly(A) tail length during the period of development studied here, using different methods (Eichhorn *et al.* 2016; Lim *et al.* 2016). This allowed us to compare the distribution of poly(A)-tail lengths of genes in our mRNA-Seq dataset as compared to all poly(A)-tail lengths of all genes reported in each of these studies. There were no significant differences between the distribution of poly(A)-tail length of our genes and of all genes reported in either the Eichhorn *et al.* 2016 or the Lim *et al.* 2016 studies (Wilcoxon text, *P* = 0.61 and *P* = 0.94 respectively). The Eichhorn *et al.* 2016 study also reported that the lengthening and shortening of poly(A)-tails over the period of development time studied (which starts earlier and ends later than our study) did not affect the mRNA abundance measurements from their oligo(dT) enriched RNA-Seq libraries. Thus, while we cannot rule out that we are recovering a biased subset of transcripts due to oligo(dT) enrichment, it seems unlikely that this method produces a substantial bias.

### PCA and clustering analyses

For these analyses, each transcript/gene level abundance was standardized to have a mean of zero and a variance of one across embryos. Statistical analysis was performed in R (R Development Core Team 2017). Principal component analysis (PCA) was performed using the *prcomp* function in R. Hierarchical clustering was performed using the *heatmap* function in R, on the standardized genes we had previously determined to be significantly differentially expressed (via the analysis described in the preceding section).

### GO and network analysis

Gene ontology analysis was performed using PANTHER (Mi *et al.* 2017), using the statistical overrepresentation test on default settings, using the GO complete annotations for biological process, molecular function, and cellular component (Table S5). We performed the analysis on the genes previously identified as significantly differentially expressed between our treatment (25%, 5%) and control (100%) groups. We ran the upregulated genes and downregulated genes separately, and compared them to a list of all genes with transcripts present at this developmental stage. This was determined by requiring >1 TPM for all replicates of either the 100% or the 5% samples. The results from this PANTHER analysis with a Bonferroni corrected p-value less than 0.05 can be found in Table S5. Visualization of this data were created using the GOplot package (Walter *et al.* 2015) in R using the GObar plotting function. Further analysis, including KEGG pathway analysis (Kanehisa *et al.* 2017) was performed in STRING (Szklarczyk *et al.* 2017).

### Fecundity Assay

To determine the effect of larval nutrition on the fecundity of flies, we first set up food dilution bottles at 5%, 25%, and 100% as reported above, with 2-4 replicate bottles for each treatment. As before, 50 eggs were placed into the food dilution bottles, allowed to hatch into larvae and pupate. Once the flies eclosed, 10 females and 10 males from each food treatment were placed into egg collection bottles (at least three replicates), and the eggs were collected twice a day for 12 days. Eggs were counted for three replicate bottles per food percentage.

### Data Availability

All sequencing data from this study are available at NCBI/GEO at accession number: GSE106308. Processed data are available under the same accession, and as supplemental tables accompanying this publication. Supplemental material available at Figshare: https://doi.org/10.25387/g3.6143183.

## Results

In order to investigate the effect of parental nutrition limitation on maternal mRNA deposition into the egg, we raised larvae in differing nutritional conditions, allowed those larvae to develop into adults, and collected their eggs for analysis. Consistent with some previous studies (Vijendravarma *et al.* 2010), we produced differences in nutrition by diluting the “standard” cornmeal food, so that larvae were supplied with either 100%, 25%, or 5% of normal food. Flies were from an Oregon-R laboratory stock of *D. melanogaster*, and the limited food treatment was restricted to the larval stage of the parents. Stage 2 embryos were selected for RNA extraction, as this stage contains only maternal RNA, and has distinct morphological characteristics to assure collection of the correct stage regardless of potential differences in development time due to treatment. Total RNA was extracted from individual embryos, three individuals per treatment, for both experimental and biological replication (see Methods), and sent for mRNA-Seq library preparation using poly(A) enrichment, and sequencing. The mRNA-Seq libraries (150bp, paired-end) were sequenced on a single lane on an Illumina HS4000 sequencer.

Resulting sequencing reads were processed (see Methods) using Kallisto (Bray *et al.* 2016) for mapping to the *D. melanogaster* transcriptome and Sleuth (Pimentel *et al.* 2017) for differential expression analysis at the transcript level and gene level. Both transcript level and gene level abundance measurements demonstrate high correlations between all samples. At the transcript level, Spearman rank correlations for transcript abundances over all transcripts compared pairwise between all samples range from 0.976-0.995 (TPMs, Table S7). For all transcript abundances at the gene level, correlations range from 0.989-0.997 (scaled reads per base, Table S7). Both the highest and lowest correlations found are within a treatment group, as the highest correlations are between two of the replicates whose mothers were raised on 100% food, and the lowest are between two replicates with mothers raised on 5% food. Thus, the differences in transcript and gene level abundance measurements between our treatment groups is relatively small as compared to the amount of transcriptomic similarity between these samples. This is perhaps unsurprising, given that these samples are embryos at the same developmental stage from the same inbred genetic background.

**Figure 1:**
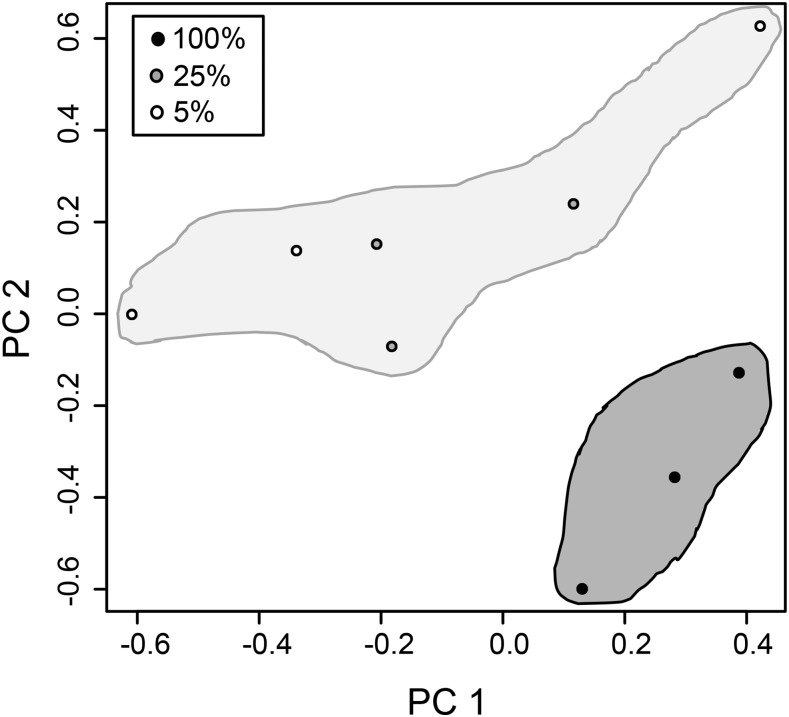
PCA plot of transcript abundances for all genes. The samples from mothers with standard food (100%, black points) group together, while the samples from mothers with reduced nutrition (25%, 5%; gray and white points) all group together.

Next, we examined changes in transcript abundance per gene over varying nutrition treatment groups and replicates using principal component analysis (PCA). Figure 1 represents the first two principal components, together representing 58% of the variance (39% and 18% respectively). Here, the samples from parents raised on 100% food group together, while all the 25% and 5% samples group together more loosely. This indicates that the changes in transcript abundance when nutrition is restricted may be similar at 25% and 5% of standard food. If we perform the PCA analysis with only the genes we identify as differentially expressed (described below), the 100% samples are clearly separated from the 25% and 5% samples (Figure S1), and the first principal component alone explains 81% of the variance.

### mRNA levels of many maternally deposited genes change with different nutritional conditions

**Figure 2:**
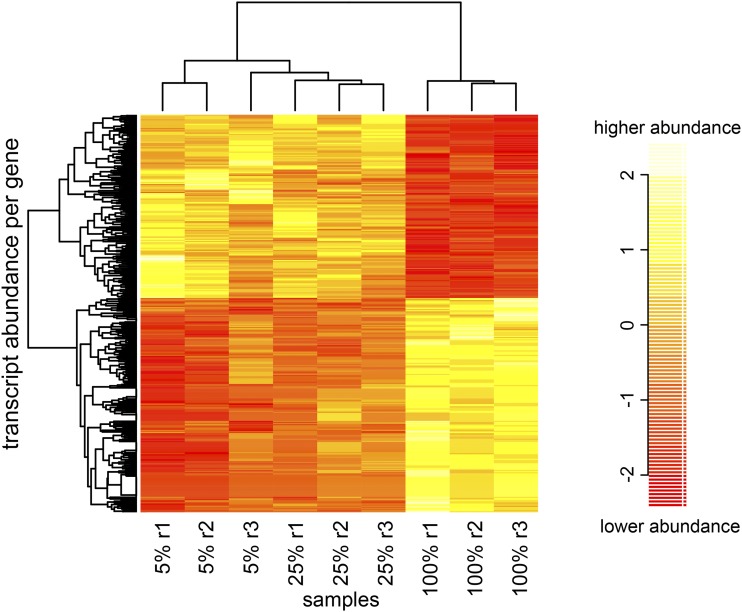
Clustering of data shows differential transcript levels of maternally deposited genes from mothers with limited nutrition. Hierarchical clustering was performed on standardized levels of genes identified as being differentially expressed between food percentage groups. Each row shows transcript abundance per gene. For the sample labels, the r1-r3 following the food percentage indicates the replicate number (1-3) for each food percentage. The 100% samples from mothers with standard food cluster together, while the 25% and 5% samples cluster together.

We identified 119 transcripts (Table S3) and 314 genes (Table S4) as differing in abundance between our different levels of nutritional limitation (25%, 5%) and controls (100%), using Sleuth (Pimentel *et al.* 2017). Almost all of the differentially expressed (DE) transcripts corresponded to genes we identified as differentially expressed in the gene-level analysis, but the gene level list is longer due to power gained by summing over transcripts in cases where genes have multiple transcripts. Of the 314 genes with significantly different transcript levels, 150 of them are represented at a lower level and 164 are more highly represented in the limited nutrition samples (25%, 5%) as compared to those whose mothers were raised on standard food. Using hierarchical clustering on genes identified as differentially expressed (Figure 2), the 100% samples group together, while one of the 5% samples is closer to the 25% samples than the other 5% samples. Figure 2 highlights the main feature of this group of genes, that the transcript level of the 100% samples is high where the 5% level is low and *vice versa* with some number of genes having an intermediate level for the 25%. That the 5% and 25% samples are so similar in transcript levels makes it difficult to determine which genes likely have an intermediate transcript level in the 25% as compared to the 5% and 100%.

### Types of maternal transcripts that change with nutrition

**Figure 3:**
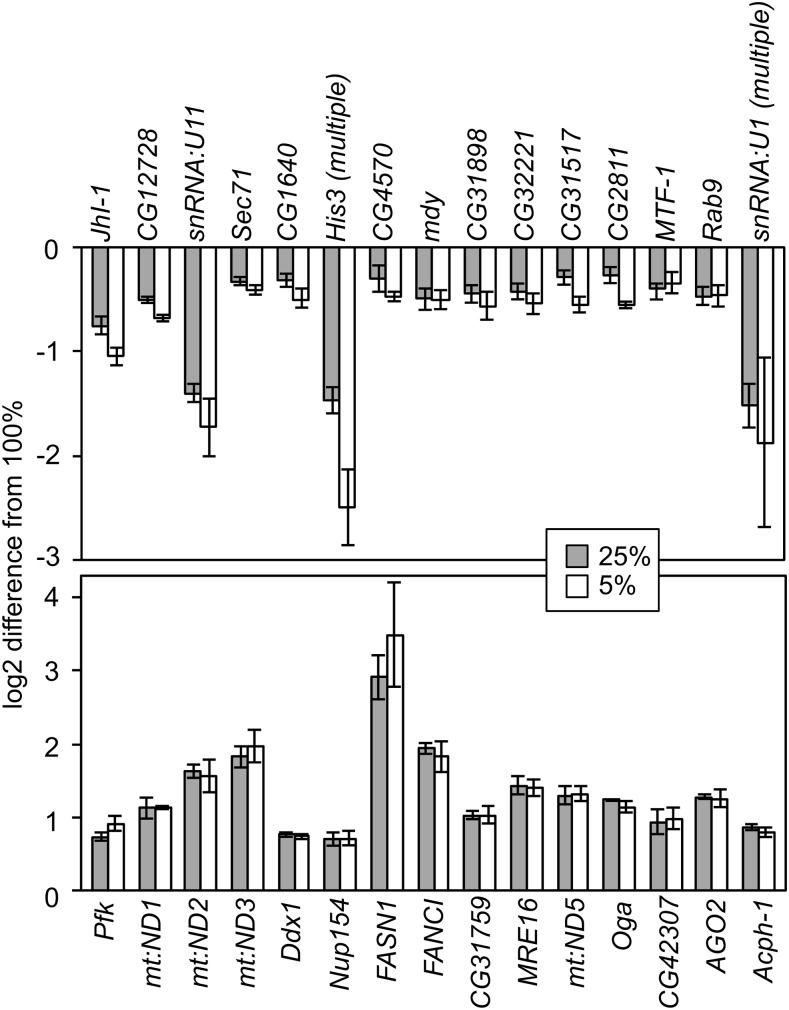
Genes most significantly differentially maternally deposited between mothers with standard nutrition and mothers with reduced nutrition. Transcript levels are the difference between the log2 scaled reads per base counts in eggs from the reduced food mothers (25%, 5%) and the standard food controls (100%). In the case of the *His3* and *snRNA:U1* genes, these are multi-copy genes with similar coding sequence, so transcripts map to multiple copies of these genes, levels here are reads mapped to a single copy (see methods).

The genes with the most significantly different transcript levels between the eggs from mothers on standard and reduced diets are represented in Figure 3. We present the top 15 differentially expressed genes (in both directions, both higher and lower transcript abundance in embryos from nutrition limited mothers) in this figure. We note that in the list of significantly differentially represented genes, genes with higher transcript abundance dominate among the most significant (*i.e.*, of the top ten differentially expressed genes, nine of them have higher transcript abundance). Among the genes with the most significantly lower transcript abundance represented in Figure 3, we do not see many of the examples of what will characterize the largest group of genes with lower transcript abundance, such as genes involved in translation (only *JhI-1* among the top 15 lower abundance genes). We do observe lower abundance of *midway (mdy)*, which is a known regulator of lipid metabolism that is critical in oogenesis; indeed, oogenesis is not completed in *mdy* mutants (Buszczak *et al.* 2002). The amount of transcription of *mdy* in our limited nutrition mothers is apparently sufficient to complete oogenesis, but this aspect of lipid metabolism is significantly less represented at the transcript level in embryos from these mothers. Among the top 15 genes with lower transcript abundance are two snRNPs, however snRNPs or other splicing factors are not significantly enriched overall. There are also a number of genes of unknown function that are observed with lower transcript abundance, some of which (*CG31898*, *CG31517*) are most highly expressed in the early embryo as compared to other developmental stages (Gelbart and Emmert 2013; Graveley *et al.* 2010).

Among the most highly differentially deposited genes that we observe to be of higher transcript abundance in eggs of nutritionally limited mothers are those involved in the electron transport chain (*mt:ND1*, *mt:ND2*, *mt:ND3*, *mt:ND5*), transport of macromolecules (*Nup154*), genes we might expect to be affected by nutrition (*Pfk*, *FASN1*, *Oga*), as well as some genes of unknown function (*CG31759*, *CG42307*). With *Phosphofructokinase* (*Pfk*) and *Fatty acid synthase 1* (*FASN1*), we find significantly higher transcript abundance of genes involved in both glycolysis and fatty acid synthesis. *O*-Linked *N*-acetylglucosamine (*O*-GlcNAc) is a post-translational modification of proteins that functions as a nutrient sensing mechanism, O*-GlcNAcase (*Oga*)* removes *O*-GlcNAc from proteins (Akan *et al.* 2016). Thus a number of our top differentially expressed genes might be expected to be affected by nutrition.

**Figure 4:**
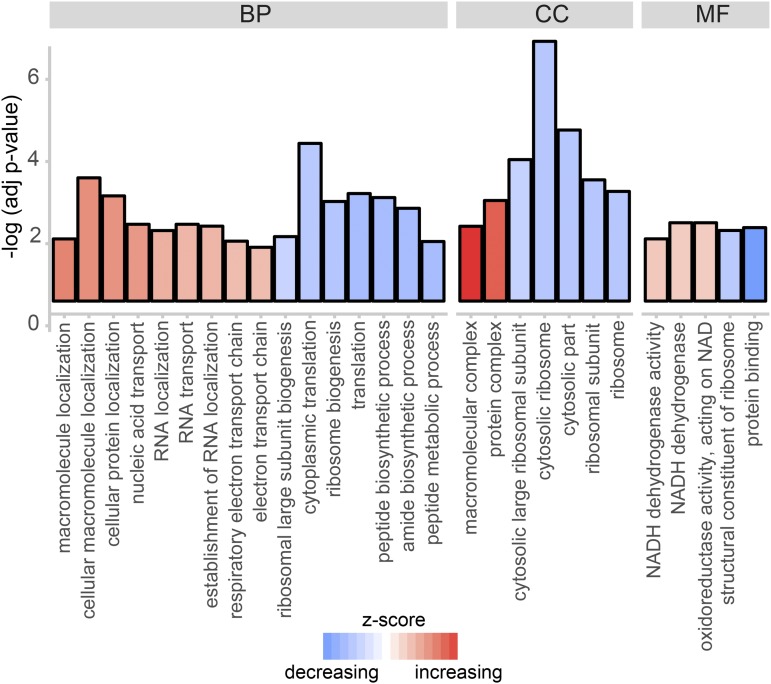
GO term analysis. Significantly enriched GO terms are pictured, grouped by category (BP: biological process, CC: cellular component, MF: molecular function). Those with increasing z-score (red) describe genes with higher transcript abundance in the nutritionally limited mothers (25% and 5% food) as compared to mothers supplied with standard nutrition, while those with a decreasing z-score (blue) are those with lower transcript abundance in nutritionally limited mothers.

**Figure 5:**
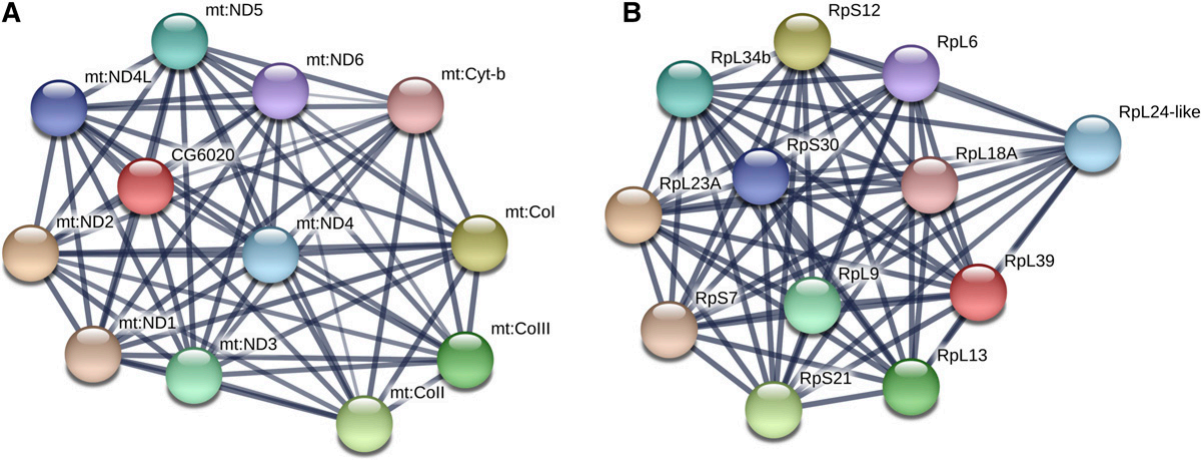
Protein network interaction diagrams for genes in most significantly enriched KEGG pathways. Edges represent protein-protein associations; line width indicates the strength of data support. A) Protein network for genes with higher transcript abundance in eggs from nutritionally limited mothers involved in the oxidative phosphorylation pathway. B) Protein network for genes with lower transcript abundance in eggs from nutritionally limited mothers involved in the ribosome.

To categorically analyze the types of genes with significantly higher or lower transcript abundance in their maternal deposition due to differences in nutrition, we performed gene ontology analysis on the differentially expressed genes using PANTHER, GO categories complete (Mi *et al.* 2017). The significantly enriched GO categories associated with our differentially expressed gene lists (higher and lower transcript abundance) as compared to the set of all genes maternally deposited in the embryo (present at stage 2 in our samples) are represented in Figure 4. Genes whose transcript levels are lower in the nutritionally limited mothers are those involved in cytoplasmic ribosomes and translation, peptide and amide biosynthetic processes, and peptide metabolism. Transcripts provided by the mother to the egg at a higher level in nutritionally limited conditions are involved in localization and transport of macromolecules in the cell and the production of ATP via the electron transport chain. These findings were further reinforced by analysis of enriched KEGG pathway ontology (Kanehisa *et al.* 2017) using STRING (Szklarczyk *et al.* 2017). As reported in Table S6, the ribosome is the only KEGG pathway term with significantly lower transcript representation in nutritionally deprived mothers, while genes in the oxidative phosphorylation, metabolic pathway, and RNA transport were all more highly represented at the transcript level. Protein network interactions for significantly differentially represented genes belonging to the most significantly differentially KEGG pathways are pictured in Figure 5.

### Comparison of changes in maternal deposition due to nutrition limitation to other studies

Since the mothers in the experimental group in this study experienced reduced nutrition during development, we ultimately want to address whether the transcripts they differentially deposit into eggs are due to either their own limitations or because they are “preparing” their offspring for nutritional limitation during their lifetimes. Because of this, we wanted to examine whether the changes in maternal transcript deposition we observe are usual cellular responses to starvation. We compare our data to previous studies. Comparing our list of 314 genes with significant changes in maternal transcript deposition due to parental nutrition limitation to the 177 genes listed under the GO term “response to starvation” for *Drosophila melanogaster* (Carbon *et al.* 2009; Ashburner *et al.* 2000), we find a non-significant (chi-squared test, *P* = 0.95) overlap of eight genes (*Bruce*, *CG9422*, *Mat89Ba*, *Nmd3*, *Ns1*, *p53*, *Pten*, *Rack1*). We also find very little overlap between our gene list and the 126 transcripts affected by starvation in a genomic study of 16 hr starved adults (Moskalev *et al.* 2015), with only two of the same genes implicated as differentially expressed (*ade3*, *Spat*; chi-squared test, *P* = 0.22). Despite the low number of genes implicated in both studies, we find higher transcript levels of some genes in the same GO categories in starved conditions in both studies, such as oxidation-reduction process and metabolic process (Moskalev *et al.* 2015). We also compared our data to two previous microarray studies, one that identified transcription changes in starved larva (Erdi *et al.* 2012), the other in starved adults (Harbison *et al.* 2005). These studies identified far more differentially expressed genes, 2819 in (Erdi *et al.* 2012) and 3451 in (Harbison *et al.* 2005). Of our differentially expressed genes, 67 or 22% of our genes were in common with the Erdi *et al.* 2012 study, and 83 or 26% of our genes were in common with the Harbison *et al.* study (Table S8; overlap was non-significant in both cases, from chi-squared test, *P* = 0.60 and *P* = 0.46, respectively). There were 16 genes on that were in common between our study and both of these microarray studies on nutrition-limited flies and larvae (Erdi *et al.* 2012; Harbison *et al.* 2005), some with known roles relating to nutrition: *ade3*, *ATPCL*, *CG11275*, *CG13631*, *CG15098*, *CG4733*, *Cyp6a17*, *GNBP3*, *Hsc70-5*, *mrt*, *Rab4*, *rdgBbeta*, *RpL13*, *Spat*, *Sps2*, *and Tif-IA*.

To expand our comparisons beyond previous studies of nutrition limitation, we also compared our list of differentially expressed genes to other pathways that we might expect to respond to starvation. We compared our data to components of the insulin/insulin-like growth factor signaling (IIS) and target of rapamycin (TOR) pathways and found again limited overlap (chi-squared test, *P* = 0.44), with only four genes (*p53*, *Pten*, *Tif-IA*, *trbl*) in common between our data and 52 core IIS/TOR components (Stanley *et al.* 2017). We noticed that some of the overlapping genes in all comparisons were general stress response factors, so we investigated the 1328 genes annotated under the GO term “response to stress” (Carbon *et al.* 2009; Ashburner *et al.* 2000). We found 45 genes in common (Table S8), which corresponds to 14% of the genes in our list (non-significant, chi-squared test *P* = 0.14). Over all comparisons to all listed studies and GO categories, we report 37 genes present in two or more of these lists in common with our data (Table S8).

In general, while there are some commonalities in genes previously implicated as differentially regulated upon starvation, part of the IIS/TOR pathways, or as part of a general stress response, our set of genes with differential transcript abundance is distinct. This is likely due to examining the effect of nutrition deprivation during development on the maternal investment in the next generation. The mothers are not currently experiencing starvation or stress themselves, but may be provisioning their offspring to face limited resources during their development. This analysis suggests that this maternal provisioning to prepare the offspring for future nutrition limitation, if occurring, does not involve many of the same genes that are currently known to respond to starvation or stress.

## Discussion

Poor environmental conditions for a parent may have profound effects on the offspring. These effects can be detrimental if they have impacts such as reducing the investment that a parent is able to make in an individual offspring. But we also sometimes observe parents preparing offspring to experience similar stresses, for example by investing more resources in a smaller number of offspring (Mousseau and Fox 1998). In Drosophila, parents with reduced nutrition produce heavier eggs (Vijendravarma *et al.* 2010; Prasad *et al.* 2003), and in some cases grow into larger adults (Valtonen *et al.* 2012). These previous studies did not measure the effect of maternal nutrition on fecundity, so we did so with the same food treatments used to produce the RNA-Seq data. We find that mothers raised with limited nutrition (5% and 25%) lay significantly fewer eggs (Wilcoxon test, *P* = 4x10^−4^; Figure S2, Table S9). Therefore, combining our result with previous work demonstrates that limiting nutrition in mothers during development results in the production of fewer but heavier eggs, from provisioning more resources to each offspring despite having fewer resources to begin with.

In this study, we asked what effect reduced nutrition in mothers would have on provisioning of mRNAs to the egg. As these maternally supplied gene products are the basis of genetic control of the organism up until zygotic genome activation, this is an opportunity for the mother to set up developmental trajectories that will influence the rest of development for this offspring. For example, perhaps the mother can supply her offspring with more of particular transcripts that can help to offset the detrimental effect of future poor nutrition on her offspring. Or, perhaps the production of transcripts (or particular transcripts) is costly to the mother. In this case, a mother who experienced limited nutrition during development would be unable to supply the same number of transcripts (or particular transcripts) to the offspring. Our data do not address whether the process of producing these transcripts is itself costly to the mother, but we did not find any systematic bias in the total amount of RNA in the egg during the extraction process to make RNA-Seq libraries. The cost to a cell of protein production is often considered to be more than transcription (Lynch and Marinov 2015), but transcription can also be as limiting under certain conditions (Kafri *et al.* 2016). Our observation of no differences in amount of total RNA indicates that transcription itself may not be limited in these mothers with reduced nutrition during their own development, likely because they were nutrient limited only during larval stage. This suggests that we can potentially view the differences in transcript levels in eggs we observe from a life-history perspective as nutrient limited mothers preparing their offspring for nutrition limitation during development.

In this study, we find that nutrition-limited mothers deposit fewer transcripts for many genes involved in translation. Many genes involved in biogenesis of cytoplasmic ribosomes, translation, and the biosynthesis and metabolism of proteins have lower transcript abundance in mothers who experienced limited nutrition as larvae. As translation is a major cost to the cell, this downregulation in translation-related transcripts may reduce the proportion of the energy devoted to these processes. One prediction of this hypothesis is that this would likely slow development time, as translation would become rate limiting. Yet in the study on which we modeled our nutritional limitation method (food dilution for parents in the larval stage) the offspring of nutrition limited mothers showed no difference in development time when raised on standard food (Vijendravarma *et al.* 2010). However, with a different nutrition limitation method, another study (Valtonen *et al.* 2012) did find a longer development time for offspring whose parents were both raised on limited food.

We found an increase in transcript levels for factors involved in the localization and transport of biomolecules. Genes involved in the transport of proteins and genes involved in the transport and localization of RNAs were particularly enriched among those with higher transcript abundance produced by these mothers. Due to previous study of the effect of nutrition on oogenesis, it is known that nutrition limitation in the mother during oogenesis leads to microtubule reorganization in early oocytes and mislocalization of mRNAs and proteins (Shimada *et al.* 2011). While the mothers in our study have adequate nutrition at the point they are undergoing oogenesis, it is possible that additional mRNAs for localization of biomolecules are transcribed as a hedge against their own future poor nutrition during oogenesis. This would imply that these mRNAs are in the egg as a result of their potential function during oogenesis. On the other hand, early embryogenesis is the time when positional information is being established for the rest of development, so perhaps additional transcripts for genes involved in transport and localization of mRNAs and proteins are supplied so that this critical process does not fail. Additionally, if transport and localization is occurring in a larger embryo, possibly for a longer period of time, more transcripts may be needed.

The other group of transcripts with higher abundance in eggs from nutritionally deprived mothers in our study relate to the electron transport chain (ETC). The number of mitochondria increase in late oogenesis (Cox and Spradling 2003; Hurd *et al.* 2016), but display low levels of activity that increases through embryogenesis following egg activation (Van Blerkom 2011; Ramalho-Santos and Amaral 2013; Dumollard *et al.* 2007). A recent study (Sieber *et al.* 2016) showed that the downregulation of insulin signaling that occurs in late oocytes results in the low activity state of mitochondria due to remodeling of the electron transport chain complexes. This results in an accumulation of glycogen late in oogenesis that is critical for the development of the egg, and that mitochondrial activity is upregulated again as embryogenesis proceeds (Sieber *et al.* 2016).

As the downregulation of ETC complexes during oogenesis is necessary for progression through oogenesis and presumably into embryogenesis, we are left with two possibilities to explain our upregulated ETC transcripts: one, that the shut down of ETC during oogenesis was not as strong as in well-fed mothers; or two, that mitochondria are active earlier in embryogenesis in eggs from nutrition-limited mothers. If the ETC was not downregulated as much during embryogenesis, this would predict that the eggs would contain less glycogen, which would be inconsistent with the idea that these mothers are better provisioning fewer eggs. Alternatively, mitochondria may become active earlier in development in the eggs from these mothers. A previous study (Tennessen *et al.* 2014) found a switch in gene expression in mid-embryonic development (∼12 hr after egg lay) to glycolytic gene expression, but in contrast to the canonical aerobic glycolytic pathway, genes involved in the TCA cycle and the electron transport chain are also upregulated. Evidence points restoration of mitochondrial membrane potential as early as blastoderm stage (Bownes’ stage 5, mitotic cycle 14) of embryogenesis (Sieber *et al.* 2016), which is after the activation of the zygotic genome. But our study examines embryos earlier stages (stage 2, >1 hr earlier than blastoderm stage begins, > 6 hr before the mid-embryonic stage discussed above), thus may represent earlier reactivation of mitochondrial activity in embryos from mothers with reduced nutrition.

### Conclusions

In this study, we demonstrate that maternal deposition of mRNA into the egg is affected by the nutritional status of the mother during her development. We characterize which transcripts are affected and what processes these transcripts are involved in. The fitness consequences of these changes in transcript representation in the egg remains to be determined, and will need to be considered in the context of the other life history traits effecting development.
